# Supplementary material for: Connexin46 Expression Enhances Cancer Stem Cell and Epithelial-to-Mesenchymal Transition Characteristics of Human Breast Cancer MCF-7 Cells
Source: Int J Mol Sci. 2021 Nov 22;22(22):12604. doi: 10.3390/ijms222212604 (PMC8624448; doi:10.3390/ijms222212604)
Supplement: Supplementary file 1 [file ijms-22-12604-s001.zip › ijms-1381877-supplementary.pdf]

Supplementary Figure S1.

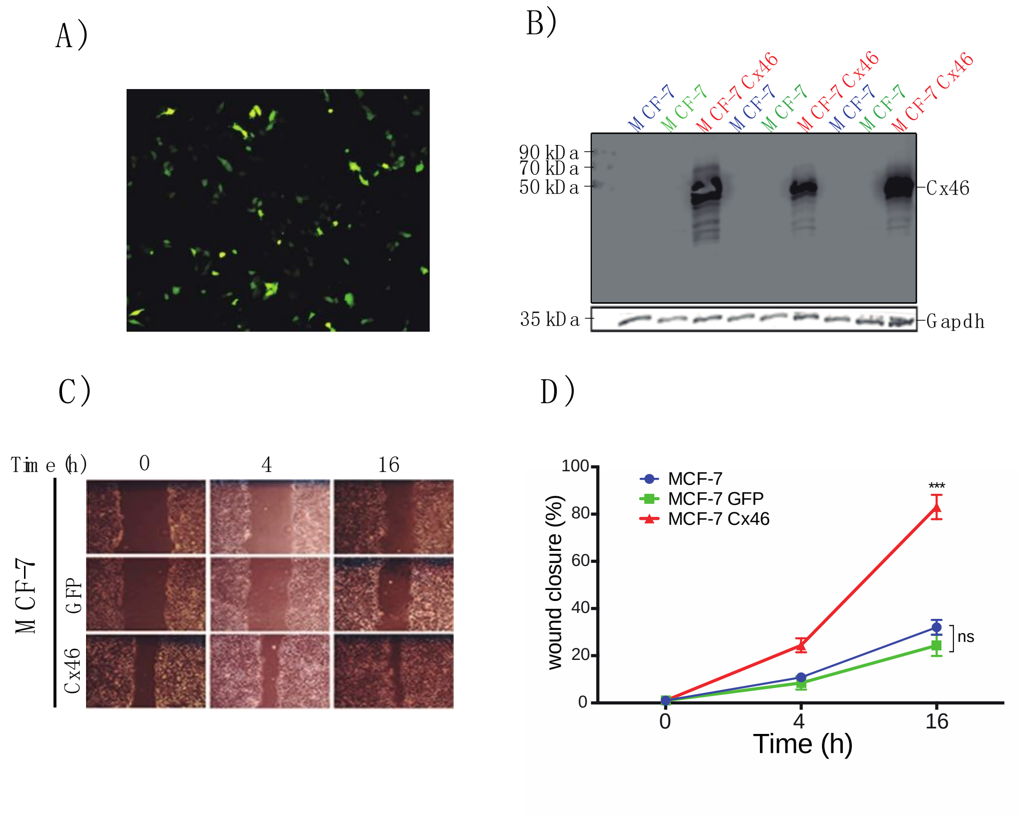

Supplementary Figure S1. Cx46 increase the migration of MCF-7 breast cancer cells similar to Cx46-GFP. (A) Representative image of MCF-7 cells transfected with GFP. (B) Western blot assay was performed to evaluate the expression of Cx46 in three different MCF-7 cell lines evaluated in triplicated using antibody against Cx46 and Gapdh as control (Santa Cruz Biotechnology; 1:2500). Protein bands were detected using Immobilon Forte western horseradish protein (HRP) substrate and visualized with a LI-COR CDigit Systems. (C) Representative images of the scratch assay at 0, 4 and 16 hours for each cell line respectively obtained at the indicated time points using a 4X objective on a Nikon inverted microscope. (D) Graphical representations of percentage of wound closure area of three different experiments represented in C, measured using the ImageJ software. The closing percentage was calculated at different times until completing the 16 hours. Data represent the means of three independent experiments +/- SEM. * denotes $p < 0.05$, ** $p < 0.01$ and *** $p < 0.001$.
